# Farther Remarks on the Treatment of Phthisis Pulmonalis

**Published:** 1790

**Authors:** William May

**Affiliations:** Extra-Licentiate of the College of Physicians, London; and Physician at Maidstone in Kent


VI. *Farther Remarks on the Treatment of Phthiſis Pulmonalis. Communicated in a Letter to Dr.* Simmons, *F. R. S. by* William May, *M. D. Extra-Licentiate of the College of Phyſicians, London; and Phyſician at Maidſtone in Kent.*

WHEN the paper on the ſubject of "a "caſe attended with ſymptoms of "phthiſis pulmonalis" was firſt publiſhed in the London Medical Journal *, it was not my intention to have proſecuted any farther the inquiry which was therein ſuggeſted reſpecting

* Vol. IX. page 268.

the the propriety of adopting a tonic mode of treatment in all cafes of true pulmonary confumption: I was not, however, without hopes that the fubject might attract the attention of fome medical man, whofe experience might correfpond with mine, and whofe authority might be fufficient to give additional weight to my obfervations on a matter of fo great profeffional and general importance. The expectations I had formed have been in a great meafure gratified. In the fecond volume of the Memoirs of the Medical Society of London, Dr. Percival, of Manchefter, has related two cafes, the fymptoms of which very nearly refembled thofe of my patient, and in which he directed a method of treatment in a great meafure fimilar to that which I have recommended, and which was attended with furprifing fuccefs *. During the interval which has paffed fince the publication of the account above mentioned I have had opportunities of obferving two or three cafes, of which I think it neceffary to give no other defcription than that they bore fo exact a fimi-

* Vide Medical Cautions and Remarks, by T. Percival, M. D. F. R. S. &c.—Memoirs of the Medical Society of London, Vol. II.

litude to that which I have already defcribed, and had fo ftrict a correfpondence with the beft nofological defcriptions of the difeafe in queftion, as left no doubt upon my mind that they were cafes of true pulmonary confumption. One of the patients was a man aged about five and thirty, whofe illnefs had been of fome months ftanding; another was a young man, upwards of twenty, who had laboured with a cough, purulent expectoration, and hectic fever, with confiderable emaciation for feveral months; the third was a young man, who was for fome weeks under the care of a fenfible and intelligent practitioner in the weft of England, and whom I faw once only. In all thefe cafes the fame method was employed as has been already related, namely, a nutrient regimen; the bark and myrrh, varioufly combined with fteel and other tonic remedies; the occafional ufe of opiates, and as much exercife as the ftate of their ftrength and other circumftances feverally permitted: in a word, a tonic regimen, in its fulleft extent, was made ufe of till the difeafe was effectually removed; and, from the lateft inquiries, I have been informed that no relapfe has taken place in either of the patients, but

 that that they continue to enjoy a good ſtate of health.

The reaſoning which I have attempted in the work alluded to is with a deſign to prove that the opinion which has hitherto obtained with reſpect to the inflammatory nature of conſumptive diſeaſes has been founded upon a miſtaken pathology *, and that upon theſe erroneous grounds the antiphlogiſtic regimen has been ſo generally, and I may add, ſo very unſucceſsfully, adminiſtered. That a ſpecies of inflammatory affection is preſent in all caſes of phthiſical diſeaſe I do not mean to deny. Without this I am ſenſible that the purulent expectoration could not be produced; but this inflammation may be of a particular or ſpecific nature, and of that kind which may require an invigorating treatment for its removal. This is the doctrine for which I contend; and, except in its application to this particular form of diſeaſe, there is certainly nothing of novelty in it. In ſcrophulous inflammations, originating in laxity and debility of the affected organs, and of the ſyſtem at large, whether the meſenteric glands, the large joints, or other parts of

* Vide London Medical Journal, Vol. IX.

the body, have been the feat of the difeafe, practitioners have found no difficulty in recommending the ufe of tonic remedies. We have the beft authority in the records of medicine with refpect to the efficacy of the Peruvian bark and cold bathing in almoft every form of fcrophulous affection. It has been owing to the want of a due diftinction being made between that active inflammatory diathefis, which confifts in an increafed tone and contractility of the vafcular fyftem, and that produced by the condition here defcribed, that an error has prevailed in practice, which appears to me fo obvious as to ftrike at firft view every obferver. Dr. Percival, with great propriety, has remarked, that in fome cafes wine and cordials are the moft powerful antiphlogiftics in nature *. He illuftrates this by ftating the method of treatment in cafes of cynanche maligna, and diforders of that clafs; and the doctrine is equally applicable to every fpecies of pyrexia in which debility appears, as is evidently the cafe in hectic fever, to be the proximate caufe of the difeafe.

* Memoirs of the Medical Society of London, Vol. II.

When it is confidered that the caufes of inflammation, and the condition of parts actually labouring under inflammatory affection, are fo widely different, as have been related above, a fact which the greateft enemies to the fyftem I would recommend will not attempt to controvert, it muft appear that the indication of cure, under thefe varieties of difeafed condition, muft alfo materially differ, and that while bleeding and an antiphlogiftic regimen, with refrigerant or debilitating medicines, are requifite in the one inftance, corroborating remedies are indifpenfably neceffary in the other. To illuftrate this, let us fuppofe a cafe of fimple ophthalmia occurring in a perfon of a found habit, with a predifpofition to an inflammatory diathefis, and the fame cafe affecting a perfon of a delicate or fcrophulous habit of body. The difeafe in either inftance may be faid to be the fame; but is the rational method of treatment the fame? Certainly not. In the former, topical, and, perhaps, general bleeding, the ufe of purgatives, with cooling and fedative applications to the affected part, will be neceffary: in the latter, not only the local affection will require the aid of tonic and aftringent applications, but the additional affiftance of the bark, and every part of of the tonic regimen may be neceffary to effect a cure. The application of this to affections of the lungs is obvious; and efpecially in the cafe under our confideration it may, I think, be urged with much force and propriety. The inflammation which accompanies the fuppuration of tubercles in the early ftages of phthifis pulmonalis I confider to be of a fcrophulous nature. The habits of perfons moft commonly, nay univerfally, affected with phthifis, the nature of the fubftance which is the feat of the inflammation, the flow progrefs of the difeafe, together with its concomitant fymptoms of emaciation and debility, all forbid the idea of any active inflammatory diathefis, and are the ftrongeft proofs that can be adduced, amounting, in my opinion, to abfolute confirmation of the oppofite ftate of the fyftem: of fuch a ftate, which, with my idea of the importance of this confideration, cannot be repeated too often, as requires the ufe of a nourifhing diet, and cordial and ftrengthening remedies.—" Ci-" bi vero," fays Celfus, " effe debent ex his " qui facile concoquuntur, maximeque alunt; " ergo vini quoque neceffarius ufus." — Dr. Cullen's account of the difeafe I have already quoted as an argument very ftrongly in favour of of my opinions refpecting it. "Debility and "emaciation of the body" certainly argue a ftate of the fyftem totally incompatible with the account of an inflammatory diathefis, as it may be collected from Dr. Cullen's Firft Lines. Indeed, from the fame work, I might adduce ftronger evidence in favour of the mode of treatment which I believe neceffary in confumptive cafes. The tenor of the chapter on phthifis pulmonalis * feems to argue an opinion of a fcrophulous diathefis accompanying, or rather giving rife to, the difeafe. The noxious acrimony which appears in one cafe, and that too a very frequent one, of phthifis, is of the fame kind, according to Dr. Cullen's opinion, with that which prevails in the fcrophula. He obferves, that a phthifis, at its ufual period, frequently attacks perfons born of fcrophulous parents; that very often, when the phthifis appears, there occur at the fame time fome lymphatic tumours in the external parts; and he has alfo found very often the tabes mefenterica joined with the phthifis pulmonalis. Dr. Cullen farther obferves, that even when no fcro-

* Vide Firft Lines of the Practice of Phyfic, Vol. II. c. iv. f. 1.

phulous phulous affection has either manifestly preceded or accompanied phthisis, this disease, however, most commonly affects persons of a habit resembling the scrophulous. He describes these as persons of a sanguine, or a sanguineo-melancholic temperament, having fine skins, rosy complexions, large veins, soft flesh, and a thick upper lip *. In all this it is evident that the phthisis pulmonalis has a very strong analogy with scrophula, if it be not absolutely a species of that disease. There are, however, those who assert that an idiopathic phthisis pulmonalis is in all instances entirely and *bona fide* a scrophulous affection. This assertion was made the subject of a thesis by my good friend and fellow graduate, Dr. Lane, and ably defended by him before the learned Professors of the University of Leyden, who have not yet relinquished the doctrine of peculiar acrimonies taught by their predecessors, and the majority of whom are still inviolably attached to the absurdities, *pace Boerhaavii memoriæ!* of the humoral pathology.

It is not totally unconnected with the subject of this letter that I here mention the

* Vide First Lines of the Practice of Physic, Vol. II, cap. iv. *passim.*

name name of Dr. Brugmans, Profeſſor of Botany at Leyden, who, in a work publiſhed ſome time ago at Gottingen, entitled "Diſſertatio "de Puogenia," has begun a reformation of the medical ſcience in that part of the continent: he has, by a train of experiments very admirably arranged, and by a ſubſequent chain of ingenious arguments, thrown conſiderable light upon the ſubject of the production of pus, and has ably controverted the doctrines of the humoral pathology with reſpect to this material ſubject. From Dr. Brugmans' experiments we are taught that an abſceſs is not to be conſidered as abſolutely neceſſary to the exiſtence of purulent matter; we are rather taught to believe, with the late Profeſſor De Haen, that pus is ſometimes formed in the blood veſſels, which, under a particular diſeaſed action, become, in fact, ſecretory organs, and from thence that it may be poured into the bronchiæ, and produce all the phenomena of phthiſis pulmonalis, without the aid of that ulceration which has been, in my opinion, erroneouſly ſuppoſed to be preſent in all caſes of the diſeaſe.

In a future letter, with which I ſhall take the liberty of troubling you on this ſubject, I propoſe pose to recur to the cases related by Dr. Percival, of Manchester, which I consider as a very respectable testimony in favour of the opinions I have ventured to offer respecting the history and treatment of phthisis pulmonalis. He has recorded these cases with a commendable candour: the brevity with which they are related cannot excite any suspicion of the possibility of fallacy, when the reputation of their author is considered, nor can their authenticity for a moment be doubted.

*Maidstone,*

July 29, 1790.

